# GPC2 deficiency inhibits cell growth and metastasis in colon adenocarcinoma

**DOI:** 10.1515/med-2022-0421

**Published:** 2022-02-14

**Authors:** Lumin Lin, Yanbin He, Zhuona Ni, Min Zhang, Jie Liu, Qianqian Mao, Bin Huang, Jiumao Lin

**Affiliations:** Department of Spleen and Stomach Diseases, The Second Affiliated Hospital of Fujian University of Traditional Chinese Medicine, Fuzhou 350003, China; Academy of Integrative Medicine, Fujian Key Laboratory of Integrative Medicine on Geriatrics, Fujian University of Traditional Chinese Medicine, Fuzhou, Fujian, 350122, China; Academy of Integrative Medicine of Fujian University of Traditional Chinese Medicine, Fuzhou, Fujian 350122, China; Key Laboratory of Integrative Medicine of Fujian Province University, Fujian University of Traditional Chinese Medicine, Fuzhou, Fujian 350122, China

**Keywords:** GPC2, colon adenocarcinoma, prognosis, tumor progression

## Abstract

Glypican-2 (GPC2) has been reported to promote tumor progression through metabolic pathways. However, the role of GPC2 in colon adenocarcinoma (COAD) remains to be further investigated. This study was designed to evaluate the role of GPC2 in COAD. Based on patients with complete clinical information and GPC2 expression from the Cancer Genome Atlas-COAD database, we found that GPC2 mRNA was highly expressed in COAD tissues, which was associated with poor prognosis and tumornode-metastasis (TNM) stage. The predicted survival probability based on GPC2 mRNA expression and TNM stage was in good agreement with the observed survival probability. Furthermore, the genes coexpressed with GPC2 in COAD tissues were significantly enriched in basal cell carcinoma, Notch signaling pathway, and Hedgehog signaling pathway. After GPC2 was decreased through transfecting short hairpin RNA of GPC2 into HCT-8 and SW620 cells, cell cycle was arrested in G0/G1 phase, proliferation was decreased, apoptosis was increased, and migration and invasion were repressed. In conclusion, decreasing GPC2 significantly inhibited proliferation, migration, and invasion, and enhanced apoptosis, which implied that GPC2 can be considered a promising therapeutic target of COAD in the future.

## 1 Introduction

Colon adenocarcinoma (COAD) is one of the most familiar severe tumors in the world [1]. As a common malignant tumor of digestive system, the incidence and mortality of COAD have increased in recent 10 years, but its deep occurrence and regulatory mechanism are still unclear, and there is a lack of effective prevention and treatment. Studying the mechanism of colon cancer development is important for the clinical treatment and prognosis of colon cancer. In recent years, a large number of studies have shown that more than 20% of patients are diagnosed with colon cancer and have distant metastasis, the most common site is the liver, and 2.1% of newly diagnosed colon cancer patients have lung metastasis [2]. Currently, the cornerstones of colon cancer treatment are surgery, neoadjuvant radiotherapy (patients with rectal cancer), and adjuvant chemotherapy (patients with stage III/IV and stage II colon cancer). The 5 years relative survival rate ranges from greater than 90% for stage I patients to greater than 10% for stage IV patients [3]. With the more and more extensive application of molecular targeted drugs in clinical practice, the study of molecular therapeutic targets has received the attention of many scholars. From a genomic point of view, COAD usually occurs with a heterogeneous series of severe diseases within the colon [4]. It has been studied that single activating mutations in the Wnt/β-catenin pathway induce the neoplastic transformation of intestinal cells [5]. Most COAD initiates the tumor development pathway by activating the Wnt pathway [6]. Some studies have explored the molecular mechanisms that play a vital role in the development of COAD, including Wnt, phosphoinositide 3-kinase, transforming growth factor-β, rat sarcoma-mitogen-activated protein kinase, and the DNA mismatch repair pathways [7,8].

Glypicans (GPCs) are heparan sulfate proteoglycans (HSPGs) usually localized at the cellular membrane. This family of proteins has the ability to regulate diverse cellular functions, including morphology and survival and differentiation. A total of six phosphatidylinositol proteoglycan members, from GPC1 to GPC6, have been found in the mammalian genome [9]. HSPGs play a part in the growth and differentiation of cells. They are connected with the occurrence and development of multiple types of tumors at multiple sites in the human body, such as lymphoma, rhabdomyosarcoma, and COAD [10]. It was reported that GPCs regulate tumor development and progression by modulating Wnt, Hedgehog, and other signaling pathways [11,12]. Earlier studies had found that glypican-2 (GPC2) and GPC3 are highly specific for tumor tissues [9]. Recently reports demonstrate that the expression of GPC2 is significantly increased in multiple childhood cancers, including neuroblastoma [13].

The expression of GPC2 correlated with the patterns of neurogenesis markers in immature neurons and GPC2 is conserved protein associated with developing of nervous system. GPC2 was reported overexpressed in cerebrospinal fluid after treatment of adult neurogenesis modulators including running wheel and fluoxetine [14]. Other scholars found that the expression of GPC2 is significantly increased in neuroblastoma an undetectable in normal tissues, including the brain, heart, lung, and kidney, indicating that GPC2 is a suitable tumor antigen in neuroblastoma [13]. GPC2 can be used as a neuroma immunotherapy target, but it has not been reported in the progression of colon cancer development.

In view of the role of GPC2 in tumor progression, we explored the role of GPC2 in colon cancer development and progression by bioinformatics analysis and cellular and molecular levels to propose new molecular markers for colon cancer treatment.

## 2 Material and methods

### 2.1 Cell culture

HCT-8 and HCT-116 were cultured in Roswell Park Memorial Institute-1640 medium (GIBCO, catalog number: 31800105, Shanghai, China) supplemented with 10% horse serum (GIBCO, catalog number: 26050070, Shanghai, China), McCoy’s 5A medium (GIBCO, catalog number: 16600108, Shanghai, China) supplemented with 10% fetal bovine serum (FBS; GIBCO, catalog No.: 10091-148, Shanghai, China), respectively. SW480, SW620, and NCM460 were cultured in Dulbecco’s modified eagle medium (GIBCO, catalog No.: 11965-092, Shanghai, China) supplemented with 10% FBS. All cell lines were obtained from Xiamen Immocell Biotechnology Co., Ltd (Xiamen, Fujian, China) and maintained in a humidified incubator at 37°C with 5% CO_2_.

### 2.2 Construction of plasmid

The pLV-sh-puro vector was used to construct plasmids of GPC2 RNA interference (shGPC2) and negative control (shNC). shGPC2-1, shGPC2-2, and shGPC2-3 were constructed based on the human *GPC2* gene. The primer sequences are shown in Table 1.

**Table 1:** The primers for constructing plasmids

Name	Sequence (5′–3′)
shGPC2-1 forward primer	CCGGATGACACCCTGGCGGATTTCTCTCGAGAGAAATCCGCCAGGGTGTCATTTTTT
shGPC2-1 reverse primer	AATTAAAAAATGACACCCTGGCGGATTTCTCTCGAGAGAAATCCGCCAGGGTGTCAT
shGPC2-2 forward primer	CCGGGCAGTATGCAGATGACTGGATCTCGAGATCCAGTCATCTGCATACTGCTTTTT
shGPC2-2 reverse primer	AATTAAAAAGCAGTATGCAGATGACTGGATCTCGAGATCCAGTCATCTGCATACTGC
shGPC2-3 forward primer	CCGGGTTTGATGTACCTGCAGGAAACTCGAGTTTCCTGCAGGTACATCAAACTTTTT
shGPC2-3 reverse primer	AATTAAAAAGTTTGATGTACCTGCAGGAAACTCGAGTTTCCTGCAGGTACATCAAAC

### 2.3 Cell proliferation analysis

HCT-8 and SW620 cells were cultured in a 96-well plate at a density of 3 × 10^3^ cells per well overnight. The cells were transfected with shGPC2 or shNC for 24, 48, 72 h. After 20 μL MTT (5 mg/mL) was added to each well, the cells were incubated at 37°C for 4 h. Subsequently, the culture supernatant in the wells was aspirated, and 150 μL dimethyl sulfoxide was added to each well to dissolve the formazan crystals. The optical density was measured at a wavelength of 490 nm using an enzyme-linked immunosorbent monitor. The data are represented in terms of mean ± standard deviation (SD) for sextuple wells.

### 2.4 Cell cycle assay

After transfected with shGPC2 or shNC for 24 h, HCT-8 and SW620 cells were harvested and fixed in 70% ethanol at 4°C overnight. After washing twice with PBS, the fixed cells were incubated in PBS containing 0.2% Triton X-100 and 10 μg/mL RNase at 37°C for 30 min. Subsequently, the cells were incubated with 20 μg/mL propidium iodide for 30 min at 25°C in the dark and analyzed using a flow cytometer. Three independent experiments were performed.

### 2.5 Cell apoptosis assay

After transfected with shGPC2 or shNC for 24 h, adherent and floating HCT-8 and SW620 cells were collected and strained using annexin V-cell apoptosis 7-AAD detection kit apoptosis detection kit (Sino Biological, Catalog number: APK10448-F) according to the manufacturer’s instruction. Subsequently, the cells were analyzed using a flow cytometer. Three independent experiments were performed.

### 2.6 Migration and invasion analysis

Cell invasion and migration were tested using transwell with or without Matrigel, respectively. After transfected with shGPC2 or shNC for 24 h, HCT-8 and SW620 cells were added to the upper chamber, and culture medium containing 10% FBS was added to the bottom of the 24-well plate. After 24 h, the cells were fixed with methanol for 30 min and stained with 0.05% crystal violet for 15–20 min. Finally, the cells were observed under microscope (Nikon A1RMP, Japan), and the results were statistically analyzed. Three independent experiments were performed.

### 2.7 Extraction of RNA and real time PCR (RT-PCR)

RNA was extracted from cells using RNA extraction kit (Takara, Catalog number: 9766) according to the manufacturer’s instruction and was reverse transcribed into complementary DNA (cDNA) using PrimeScript™ RT Master Mix (Takara, Catalog number: RR036A). Subsequently, RT-PCR was performed using the obtained cDNA and TB Green^®^ Fast qPCR Mix (Takara, Catalog number: RR430A). The target gene sequences were obtained from the National Center for Biotechnology Information database to design the primers, which are shown as follows: GPC2 forward primer: 5′-GGTTCGTGGCTGTCTCAGCAG-3′, GPC2 reverse primer: 3′-GCAGGTACATCAAACCCTCCGA-5′, RNA18SN5 forward primer: 5′-ACCCGTTGAACCCCATTCGTGA-3′, and RNA18SN5 reverse primer: 3′-GCCTCACTAAACCATCCAATCGG-5′. The data are expressed as the mean ± SD of three independent experiments.

### 2.8 Western blotting

The total protein of the cells was extracted using ice-cold radio-immunoprecipitation assay buffer (Beyotime, catalog number: P0013C) and quantified with bicinchoninic acid protein concentration assay kit (Beyotime, catalog number: P0012S). After separated by using 10% sodium dodecyl sulfate polyacrylamide gel electrophoresis gel, the proteins were transferred onto polyvinylidenefluoride membranes, which were subsequently incubated with 5% skimmed milk at 25°C for 2 h. The membrane was incubated with GPC2 antibody (Biorbyt, catalog number: orb157203), PTCH1 antibody (Biorbyt, catalog number: orb538088), PTCH2 antibody (Biorbyt, catalog number: orb416229), GLI1 antibody (Proteintech, catalog number: 66905-1-Ig), NOTCH1 antibody (Biorbyt, catalog number: orb256723), HES1 antibody (Biorbyt, catalog number: orb36445), DLL4 antibody (Biorbyt, catalog number: orb97478), or glycerine aldehyde phosphate dehydrogenase antibody (Proteintech, catalog number: 10494-1-AP) at 4°C overnight, followed by horseradish peroxidase (HRP) conjugated goat anti-rabbit IgG(H + L) (Proteintech, Catalog number: SA00001-2) or HRP-conjugated goat anti-mouse IgG(H + L) (Proteintech, Catalog number: SA00001-1) at 25°C for 2 h. Finally, Super Sensitive ECL Solution (Beijing Bai’aolaibo Technology Co., Ltd, Catalog number: MT0076) was used to display the results. The experiments were performed thrice independently.

### 2.9 Bioinformatic analysis

The transcriptome profiling data of 41 normal and 469 COAD tissues were downloaded from The Cancer Genome Atlas (TCGA)-COAD. GPC2 expression data in these unpaired samples were further extracted and analyzed using the limma package (https://bioconductor.org/packages/limma (version 3.8)) in R software (version 3.5.1). GPC2 expression data in 160 paired samples were obtained from Gene Expression Omnibus (GEO, GSE87211) and analyzed using The Perl Programming Language (version 5.30.0).

The associations between GPC2 expression and living status, disease status, history of colon polyps, TNM stage, tumor size, or lymph node metastasis were determined using The Perl Programming Language (version 5.30.0).

The overall survival (OS), defined as the time from diagnosis to death or last examination, was the primary endpoint of this study. Disease-free interval (DFI) is the time from the start of surgical treatment to tumor recurrence, metastasis, or the development of tumor-related disease. According to the median value of GPC2 expression, the patients with hepatocellular carcinoma were divided into two groups, high and low expression groups, to analyze OS or DFI of total patients, patients with TNM stage I or II COAD, or patients with TNM stage III or IV COAD by Kaplan–Meier (KM) survival curves plotted in the R survival package (version 3.1-12). The receiver operating characteristic (ROC) curve was drawn, and the area under the curve (AUC) value was calculated to evaluate the ability of GPC2 to identify patients with COAD using SPSS software (https://en.softonic.com/download/spss (version 21.0)). Nomogram models were constructed by combining gene *GPC2* with TNM stage information, which was used to predict the survival rate of patients in 1, 3, and 5 years, and calibration curves were drawn to test the accuracy of the model.

Genes coexpressed in COAD tissue with GPC2 were download from cBioPortal (http://www.cbioportal.org/). Genes with Spearman’s Correlation absolute value greater than 0.3 and *q*-value less than 0.05 were selected to analyze protein–protein interactions (PPI) on the STRING website (https://string-db.org/).

Gene set enrichment analysis (GSEA) was used to analyze the possible regulatory pathways of GPC2 in COAD. A nominal *P* < 0.05 and a false discovery rate (FDR) <25% were considered to be statistically significant in enriched gene sets analysis.

### 2.10 Statistical analysis

Experimental data were analyzed using SPSS 22.0. Mann–Whitney test and Wilcoxon matched-pairs signed rank test were performed for nonparametric data between two groups and for nonparametric data between matched samples. Student’s *t*-test was used for the comparison between two groups, and one-way analysis of variance followed by Tukey’s test were used for comparison among multiple groups. Log-rank test was used for statistical analysis of KM survival curves. The level of statistical significance was set at *P* < 0.05.

## 3 Results

### 3.1 The mRNA level of GPC2 is significantly upregulated in COAD tissues

**Figure 1:**
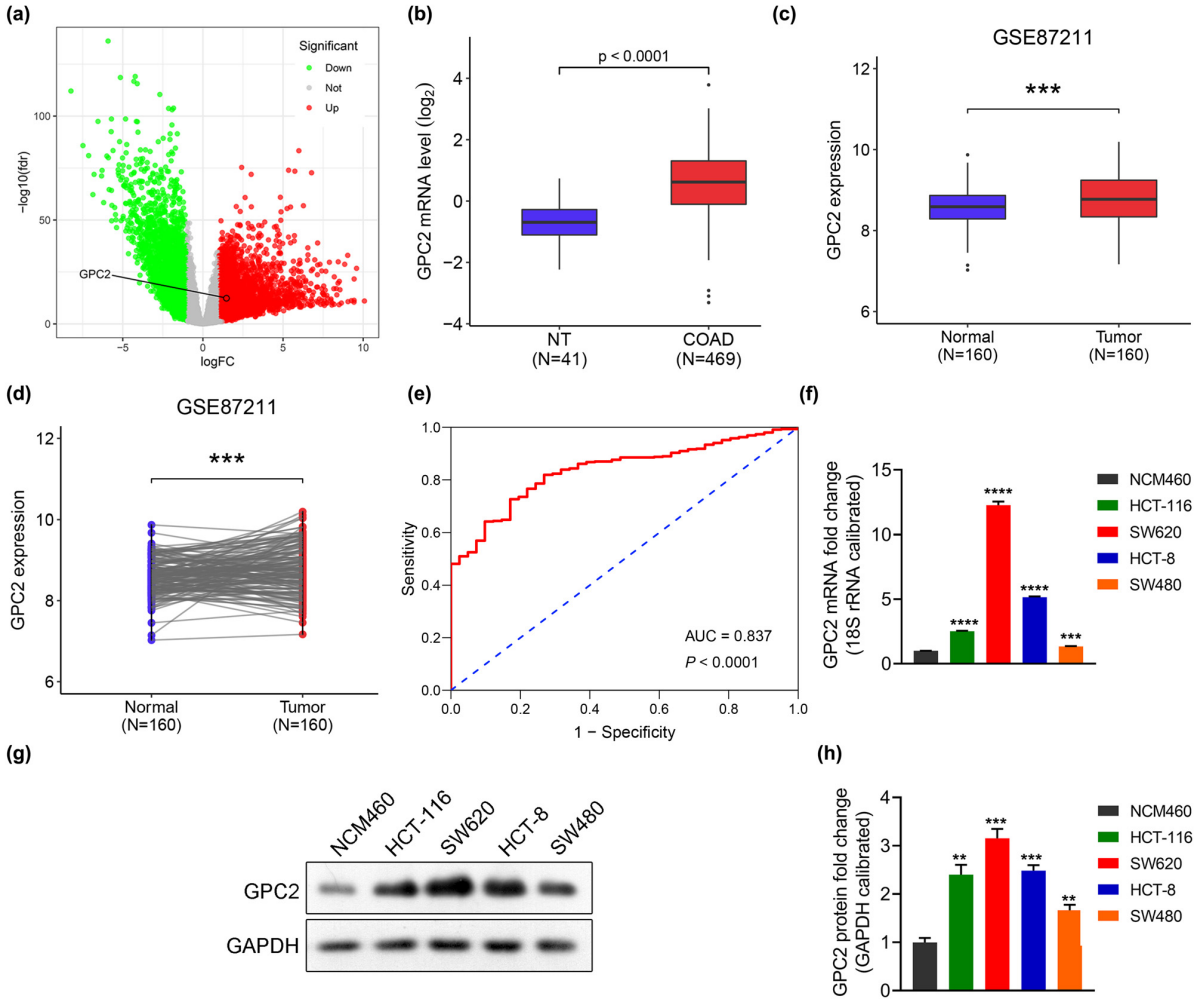
The mRNA level of GPC2 is significantly upregulated in COAD tissues: (a) volcano plot of gene expression in patients with COAD, (b) the difference in GPC2 expression in nontumor tissues (*n* = 41) and COAD tissues (*n* = 469) was analyzed, (c) the difference in GPC2 expression in nontumor tissues (*n* = 160) and COAD tissues (*n* = 160) from GEO was analyzed, (d) differential expression of GPC2 in adjacent normal tissues (*n* = 160) and tumor tissues (*n* = 160) from GEO, (e) validation of the diagnostic value of GPC2 upregulation in COAD using an ROC curve, (f) the mRNA level of GPC2 in COAD cell lines was detected using RT-PCR (*n* = 3), and (g and h) Western blotting was used to detect the protein level of GPC2 in COAD cell lines (*n* = 3). logFC: log2 fold change, NT: not tumor, COAD: colon adenocarcinoma, ***P < 0.001, ****P < 0.0001.

Data mining was performed using TCGA and GEO databases. The data were analyzed using edgeR (https://www.bioconductor.org/packages/edgeR/ (version 3.30.3)) to draw a volcano plot with a threshold of |log2 fold change (FC)| > 1 and FDR <0.05. A total of 3,827 genes were significantly upregulated (red dots) and 2,730 genes were significantly downregulated (green dots; Figure 1a). The gene of interest, *GPC2* (log2 FC = 1.471, FDR = 4.65 × 10^−13^), was selected for further analysis. The result showed that the transcription level of GPC2 in COAD tissues was significantly upregulated (Figure 1b and c). The RNA level of GPC2 in COAD tissues was significantly increased compared with the corresponding adjacent normal tissues (Figure 1d). Next, the diagnostic value of GPC2 for COAD was measured using the ROC curve, and the AUC was calculated. The ROC curve showed that GPC2 could be used to easily distinguish COAD tissues from normal tissues with an AUC = 0.837, and a *P* < 0.0001, which validated the diagnostic value of GPC2 upregulation for COAD (Figure 1e). The mRNA and protein expression of GPC2 was analyzed in NCM460, HCT-8, HCT-116, SW620, and SW480 cell lines using RT-PCR and Western blotting, respectively, and the results showed that GPC2 expression was the highest in SW620 cells (*P* < 0.001), followed by that in HCT-8 cells (*P* < 0.001; Figure 1f–h). Therefore, SW620 and HCT-8 cells were selected for subsequent *in vitro* validation tests.

### 3.2 High transcription levels of GPC2 suggest a poor prognosis in patients with COAD

**Figure 2:**
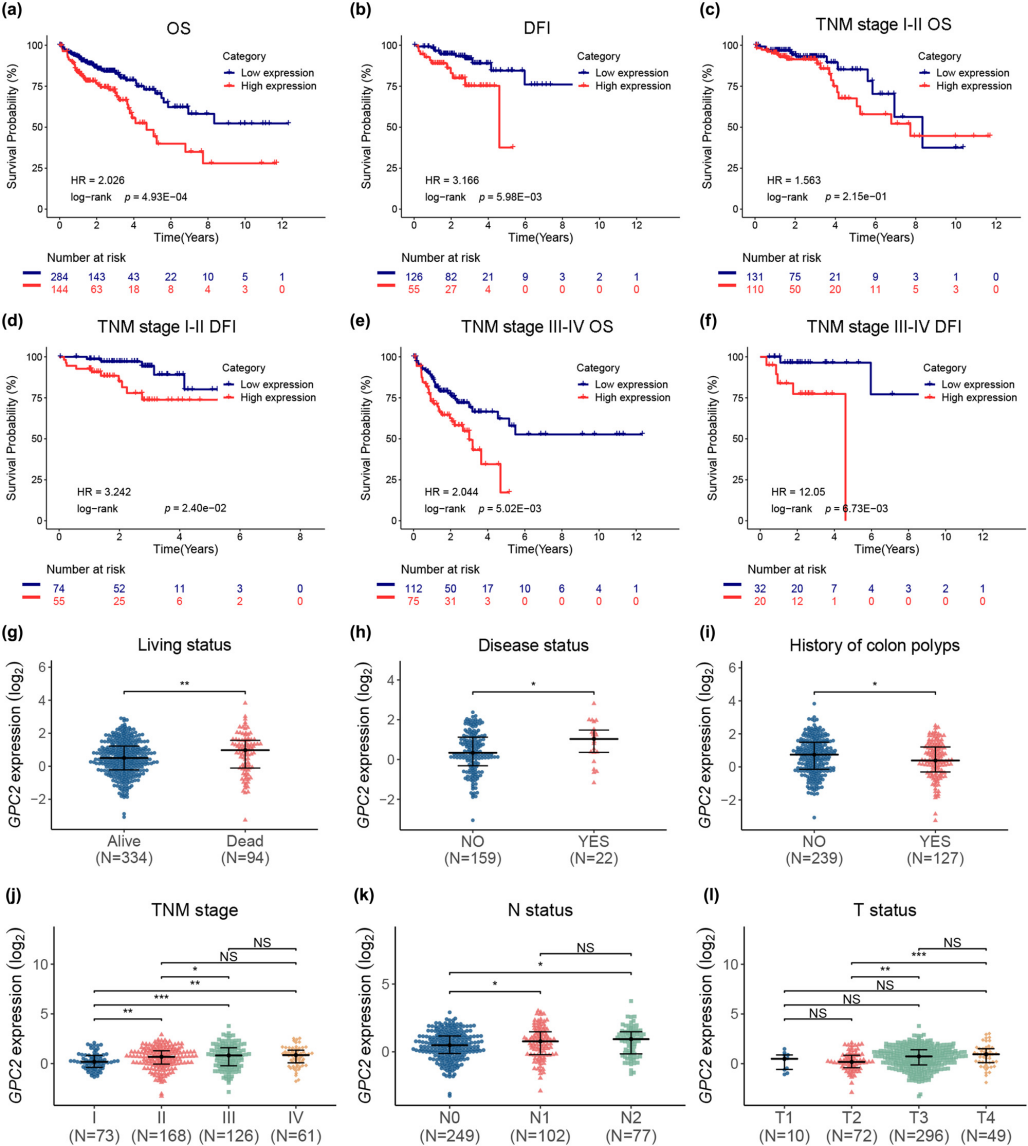
High transcription levels of GPC2 suggest a poor prognosis in patients with COAD: (a and b) correlation between GPC2 expression and the OS (a; low expression: *n* = 284; high expression: *n* = 144) or DFI (b; low expression: *n* = 126; high expression: *n* = 55) of total patients with COAD was analyzed using KM analysis, (c–f) the relationship between GPC2 level and patients’ OS (c and e) and DFI (d and f) was analyzed by KM curve based on TNM stage. TNM stage I–II OS: Low expression: *n* = 131; High expression: *n* = 110; TNM stage I–II DFI: Low expression: *n* = 74; High expression: *n* = 55; TNM stage III–IV OS: Low expression: *n* = 112; High expression: *n* = 75; TNM stage III–IV DFI: Low expression: *n* = 32; High expression: *n* = 20. (g–l) Comparison of the GPC2 expression in different groups with different living status (g; alive: *n* = 334; dead: *n* = 94), disease status (h; No: *n* = 159; Yes: *n* = 22), history of colon polyps (i; No: *n* = 239; Yes: *n* = 127), TNM stage (j; I: *n* = 73; II: *n* = 168; III: *n* = 126; and IV: *n* = 61), N status (k; N0: *n* = 249; N1: *n* = 102; and N2: *n* = 77), and T status (l; T1: *n* = 10; T2: *n* = 72; T3: *n* = 296; and T4: *n* = 49). OS: overall survival, DFI: disease-free interval. NS: not significant, *P < 0.05, **P < 0.01, ***P < 0.001.

According to the median value of GPC2 expression, the patients were divided into two groups, high and low expression, and the correlation between GPC2 expression and OS or DFI in patients with COAD was measured using KM survival analysis. The results showed that patients with high GPC2 expression exhibited a poor OS and DFI (Figure 2a and b). Among the patients at COAD TNM stage Ⅰ and stageⅡ, patients with high COAD mRNA levels had poor DFI than those with low COAD mRNA levels, whereas COAD mRNA levels had no significant effect on the patients’ OS (Figure 2c and d). Among patients at COAD TNM stage III and IV, patients with high COAD mRNA levels had worse DFI and OS than those with low COAD mRNA levels (Figure 2e and f). The transcription level of GPC2 in the COAD tissues of the dead patients was higher than that of the alive patients, and GPC2 transcriptional levels were higher in patients with disease than in patients without disease, while GPC2 transcriptional levels were lower in patients with a history of colonic polyps than in patients without a history of colonic polyps (Figure 2g–i). Moreover, the transcription level of GPC2 in the COAD tissues of the patients at advanced TNM stage was higher than that of the patients at early TNM stage, the GPC2 transcription level of patients with lymph node metastasis was higher than that of patients without lymph node metastasis, and the GPC2 transcription level in COAD tissue of patients with advanced T stage was higher than that of patients with early T stage (Figure 2j–l). Together, high level of GPC2 mRNA was associated with the progression of COAD and indicated poor prognosis.

### 3.3 Evaluation of the prognostic value of GPC2 in patients with COAD based on nomograms

**Figure 3:**
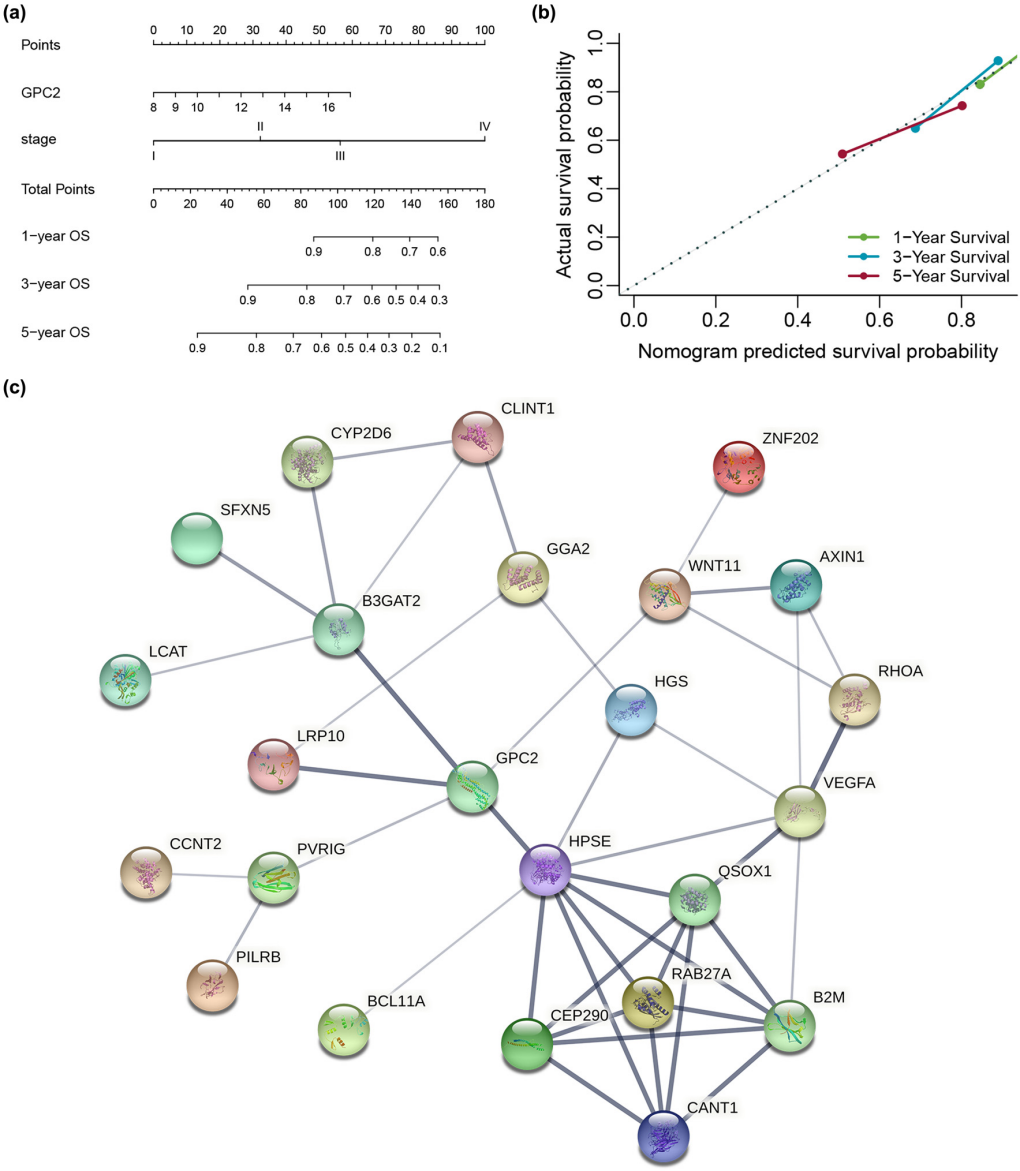
Analysis of the prognostic value of GPC2 in patients with COAD and GPC2-related PPI network: (a) postoperative prognostic nomogram for patients with COAD, (b) the calibration curve of the nomogram for predicting the OS at 1, 3, and 5 years, and (c) GPC2-related PPI network. OS: overall survival.

A nomogram model was constructed by combining the target gene GPC2 with TNM stage information to predict the 1, 3, and 5 years survival rate of patients, and the calibration curve was drawn to test the model accuracy. The results showed good consistency between the predicted survival probability and the observed survival probability. The C-index of the COAD nomogram for OS prediction was 0.731 (95% CI 0.701 – 0.761, *P <* 0.05, Figure 3a and b).

### 3.4 Analysis of PPI network

Genes with an absolute value of Spearman’s correlation higher than 0.3 for the coexpression with *GPC2* gene were selected from the TCGA-COAD dataset on the website of cBioPortal (https://www.cbioportal.org/), and then STRING (https://string-preview.org/) was used to make PPI network diagram. Based on the information of STRING protein query in the public database, GPC2 has a higher degree of connectivity. This analysis indicates that five proteins (LRP10, HPSE, B3GAT2, PVRIG, and WNT11) have the potential to directly interact with GPC2 (Figure 3c).

### 3.5 Prediction of pathways involved in GPC2

**Figure 4:**
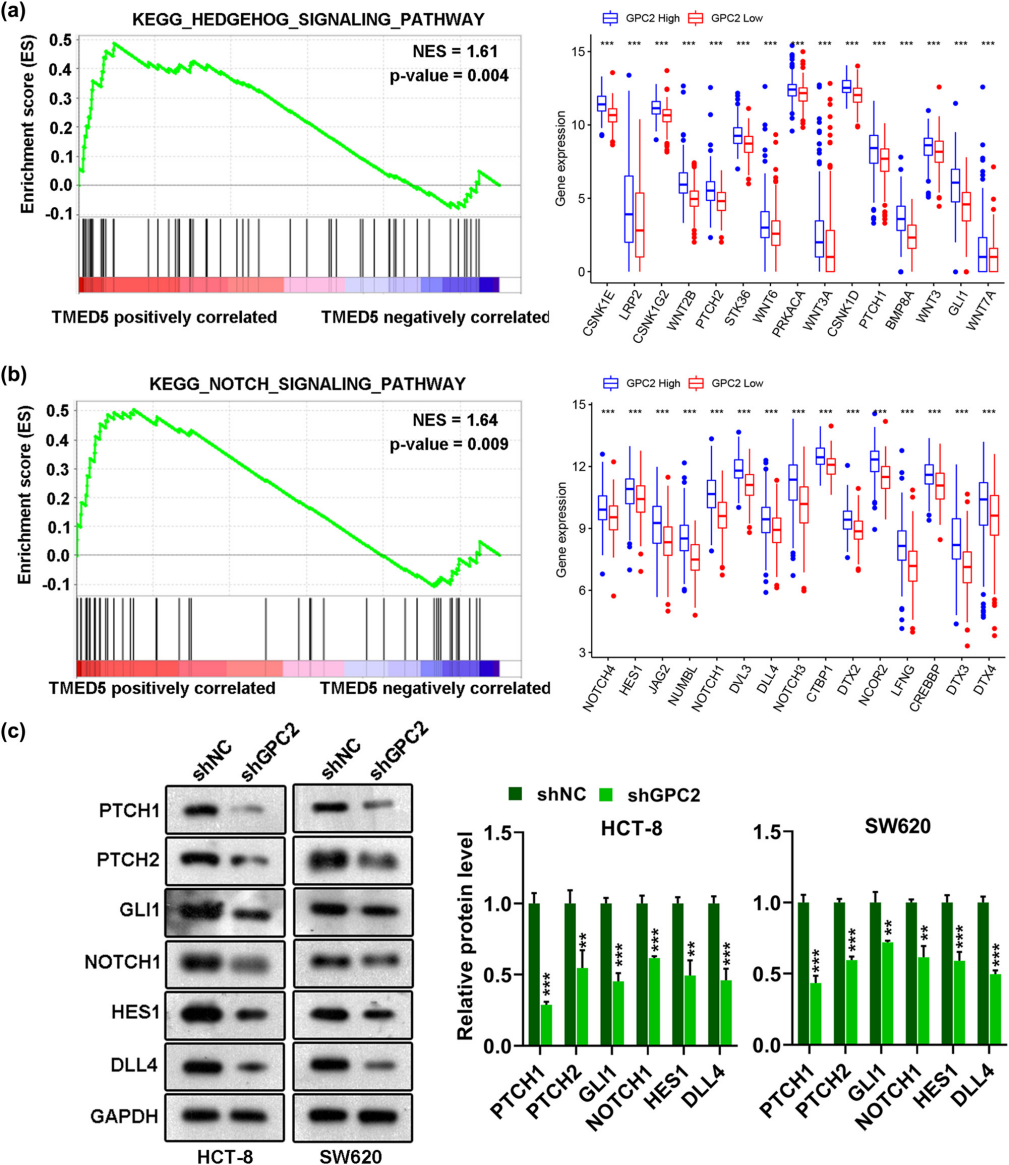
Prediction of pathways involved in GPC2: (a and b) the genes co-expressed with GPC2 in COAD tissues were enriched in (a) notch signaling and (b) hedgehog signaling and (c) After transfection of shNC, shGPC2 into HCT-8 and SW620 cells, the protein levels of PTCH1, PTCH2, GLI1, NOTCH1, HES1, and DLL4 were detected by Western blotting (*n* = 3). KEGG: Kyoto Encyclopedia of Genes and Genomes. **P < 0.01, ***P < 0.001.

GSEA was used to explore the potential biological functions of GPC2 to compare the low expression group with the high expression group in TCGA database. The results showed that genes positively related to GPC2 were enriched in “Notch signal” and “hedgehog signal” pathways (all *P* < 0.05; Figure 4a and b). Moreover, silencing GPC2 reduces the protein levels of related molecules in the “Notch signal” (NOTCH1, HES1, and DLL4) and “Hedgehog signal” (PTCH1, PTCH2, and GLI1) pathways (all *P* < 0.01, Figure 4c). These data suggest that GPC2 levels are correlated with Notch signal and Hedgehog signal pathways in COAD.

### 3.6 Decreasing GPC2 inhibits proliferation and promotes apoptosis

**Figure 5:**
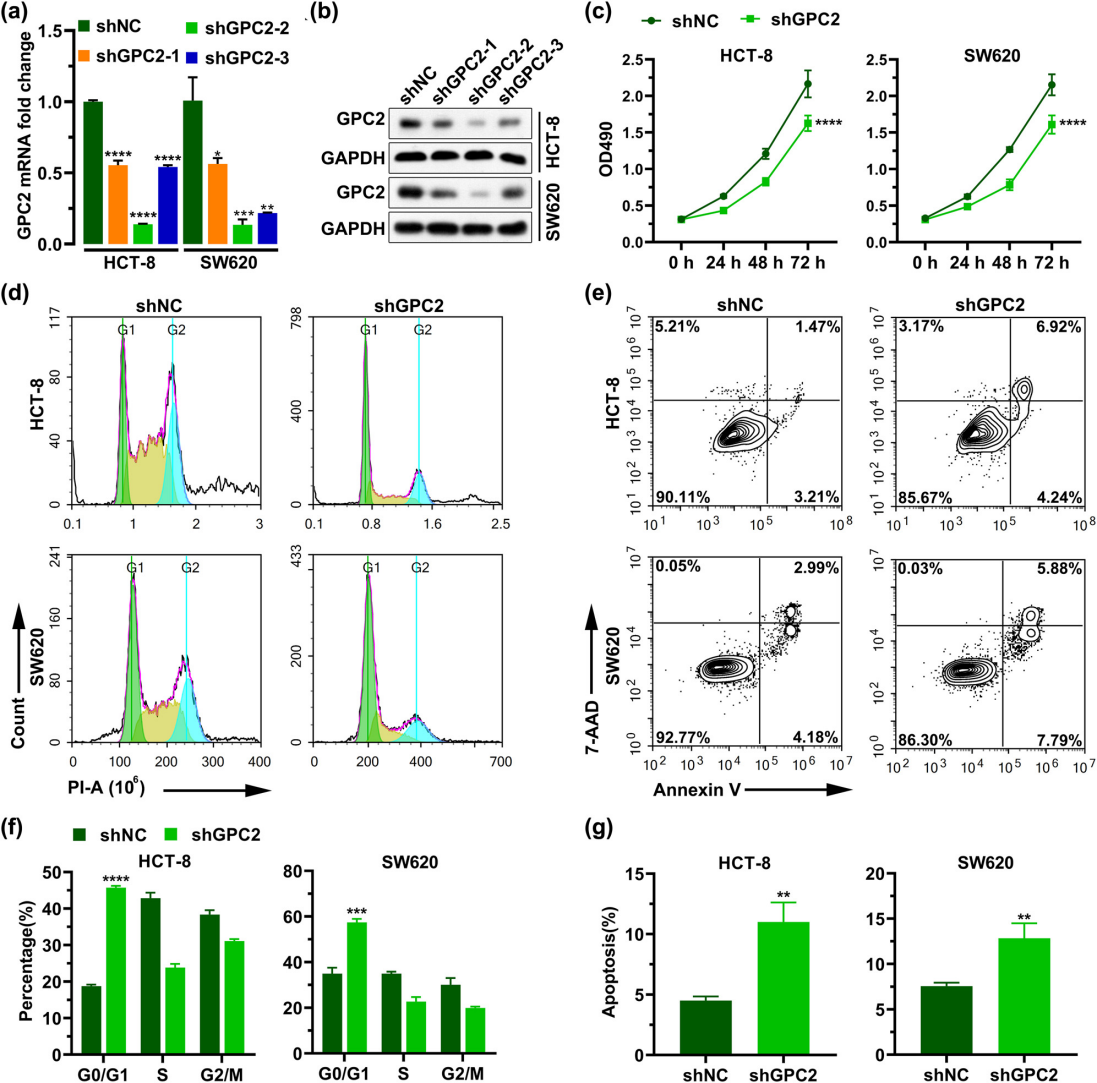
Decreasing GPC2 inhibits proliferation and promotes apoptosis in HCT-8 and SW620 cells: (a and b) after transfection of shNC, shGPC2-1, shGPC-2, and shGPC2-3 in HCT-8 and SW620 cells, the level of GPC2 was detected by RT-PCR (a; *n* = 3) and (b) Western blotting, (c) MTT assay was used to analyze the effect of silencing GPC2 on cell proliferation (*n* = 6), (d and f) Flow cytometry was used to analyze the cell proportion of different cell cycles after GPC2 silencing (*n* = 3), (e and g) Annexin V/7-AAD Apoptosis Kit was used to detect the proportion of apoptosis in HCT-8 and SW620 cells after GPC2 silencing (*n* = 3). *P < 0.05, **P < 0.01, ***P < 0.001, ****P < 0.0001.

The COAD cells HCT-8 and SW620 were divided into shNC, shGPC2-1, shGPC2-2, and shGPC2-3 groups, and the *GPC2* expression levels in each group were analyzed using RT-PCR and Western blotting. The results showed that mRNA and protein level of GPC2 in the shGPC2 groups were significantly decreased compared to those in the shNC group (Figure 5a and b). The proliferation was analyzed using MTT assay, and the results showed that the cell proliferation was significantly reduced in the shGPC2 group (Figure 5c). After GPC2 was silenced, the proportion of cells in the G0/G1 phase increased, whereas the proportion of cells in the S phase and G2/M phase decreased, indicating that the silencing of GPC2 resulted in cell arrest in the G0/G1 phase that prolonged the cell cycle, slowed down the cell division and inhibited the cell growth (Figure 5d and f). In addition, silencing GPC2 significantly increased the apoptosis rate (*P* < 0.01; Figure 5e and g).

### 3.7 Downregulating GPC2 suppresses migration and invasion

**Figure 6:**
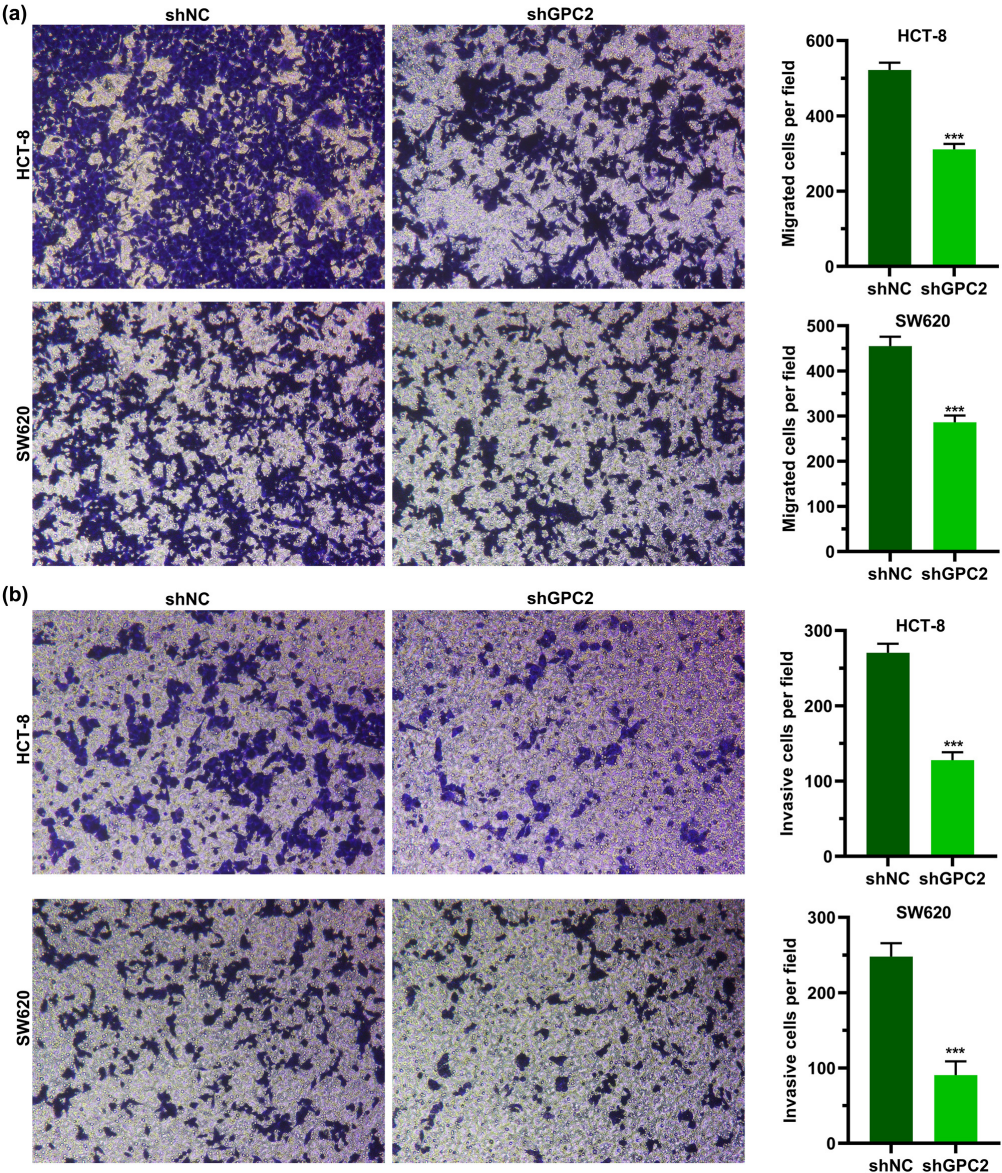
Downregulating GPC2 suppresses migration and invasion HCT-8 and SW620 cells: (a and b) the effect of GPC2 on (a) migration or (b) invasion of HCT-8 and SW620 cells was tested using transwell assay (*n* = 3). ***P < 0.001.

Next, the results of transwell assay showed that after reducing the expression of GPC2 in HCT-8 and SW620 cells, the number of migrating or invading cells was significantly decreased, which indicated that downregulation of GPC2 inhibited cell migration and invasiveness (Figure 6a and b).

## 4 Discussion

COAD is the third most common cancer in the world, and a high-fat and low-fiber diet promotes colitis and cancer development [15,16]. Patients have a good prognosis with a 5 years OS rate of 91% if detected and treated early. As the disease progresses, the survival rate significantly decreases, and the 5 years OS rate of patients with distant metastasis is 12% [17]. Therefore, better biomarkers need to be identified to open up new approaches for targeted therapy. In this study, we found that GPC2 is upregulated in COAD tissues. In addition, high levels of GPC2 indicate a poor prognosis for COAD patients, and the construction of a nomogram model combined with GPC2 and TNM staging information shows that there is a good agreement between the predicted survival probability and the observed survival probability. These results indicate that GPC2 has the potential to become a biomarker of COAD.

As the role of GPCs in tumor matrix remodeling, tumor microenvironment, regulation of tumor cell–matrix interaction, and tumor cell signal transduction has been revealed, GPCs have been proved to play an important role in the tumor development and can be used as a valuable target for the treatment of various tumors [18,19,20,21,22]. GPCs include GPC1, GPC2, GPC3, GPC4, GPC5, and GPC6 [23]. GPC1 is upregulated in pancreatic cancer, esophageal cancer, and prostate cancer, and it promotes the proliferation and movement of esophageal cancer cells by PTEN/Akt/β-catenin pathway [24,25,26]. High levels of GPC3 in serum have been shown to be a marker for hepatoblastoma and hepatocellular carcinoma [26,27]. Overexpression of GPC5 may accelerate the tumor progression of lymphoma and may enhance the interaction between Patched 1 and Hedgehog signaling in rhabdomyosarcoma [26]. GPC2 plays an important role in neural cell adhesion and neurite growth, and researchers have found that GPC2 is a powerful candidate target for immunotherapy in childhood cancer [28]. In addition, investigators demonstrated that GPC2 is required for neuroblastoma proliferation and developed a GPC2-targeting antibody-drug conjugate with significant cytotoxicity against highly expressing GPC2 neuroblastoma cells [9]. In this study, we found that GPC2 is upregulated in COAD tissues and is highly expressed in COAD cells SW620 and HCT-8. Moreover, silencing GPC2 inhibited the proliferation, migration, and invasion of SW620 and HCT-8 cells, and promoted cell apoptosis, indicating that GPC2 may play a carcinogenic role in the progression of COAD.

In addition, an existing study has shown that GPC2 knockdown inhibits the activation of the Wnt/β-catenin pathway [29]. Our results reveal that GPC2 may interact directly with five proteins directly involved in metabolic regulation and signal transduction (LRP10, HPSE, B3GAT2, PVRIG, and WNT11). The genes positively correlated with GPC2 were mainly enriched in the “Notch signaling” and “Hedgehog signaling” pathways. These findings suggest that GPC2 may be involved in the regulation of multiple signaling pathways.

In summary, the upregulating GPC2 in COAD tissues and cells is associated with the progression of COAD and indicates a poor prognosis. GPC2 may interact directly with LRP10, HPSE, B3GAT2, PVRIG, and WNT11 and may have a positive correlation with the “Notch signal” and “Hedgehog signal” pathways. Moreover, GPC2 promotes the proliferation, migration, and invasion of SW620 and HCT-8 cells and inhibits cell apoptosis. These data indicate that GPC2 may play a role in promoting the progression of COAD and has the potential to become a promising COAD treatment target.
